# Modulation of Early Host Innate Immune Response by an Avipox Vaccine Virus’ Lateral Body Protein

**DOI:** 10.3390/biomedicines8120634

**Published:** 2020-12-19

**Authors:** Efstathios S. Giotis, Stephen M. Laidlaw, Susanna R. Bidgood, David Albrecht, Jemima J. Burden, Rebecca C. Robey, Jason Mercer, Michael A. Skinner

**Affiliations:** 1 Section of Virology, School of Medicine, St Mary’s Campus, Imperial College, London W2 1PG, UK; stephen.laidlaw@kennedy.ox.ac.uk (S.M.L.); beccarobey@hotmail.com (R.C.R.); m.skinner@imperial.ac.uk (M.A.S.); 2 School of Life Sciences, University of Essex, Colchester C04 3SQ, UK; 3 Medical Research Council-Laboratory for Molecular Cell Biology, University College London, Gower Street, London WC1E 6BT, UK; s.bidgood@ucl.ac.uk (S.R.B.); david.albrecht@mpl.mpg.de (D.A.); j.burden@ucl.ac.uk (J.J.B.); jason.mercer@ucl.ac.uk (J.M.)

**Keywords:** fowlpox virus, interferon, lateral bodies, nuclear localisation signal, microarrays

## Abstract

The avian pathogen fowlpox virus (FWPV) has been successfully used as a vaccine vector in poultry and humans, but relatively little is known about its ability to modulate host antiviral immune responses in these hosts, which are replication-permissive and nonpermissive, respectively. FWPV is highly resistant to avian type I interferon (IFN) and able to completely block the host IFN-response. Microarray screening of host IFN-regulated gene expression in cells infected with 59 different, nonessential FWPV gene knockout mutants revealed that FPV184 confers immunomodulatory capacity. We report that the *FPV184*-knockout virus (FWPVΔ184) induces the cellular IFN response as early as 2 h postinfection. The wild-type, uninduced phenotype can be rescued by transient expression of *FPV184* in FWPVΔ184-infected cells. Ectopic expression of *FPV184* inhibited polyI:C activation of the chicken IFN-β promoter and IFN-α activation of the chicken Mx1 promoter. Confocal and correlative super-resolution light and electron microscopy demonstrated that FPV184 has a functional nuclear localisation signal domain and is packaged in the lateral bodies of the virions. Taken together, these results provide a paradigm for a late poxvirus structural protein packaged in the lateral bodies, capable of suppressing IFN induction early during the next round of infection.

## 1. Introduction

Poxviruses are large, enveloped, double-stranded DNA viruses capable of causing disease in mammals, birds and insects. Binding and entry of poxviruses into vertebrate cells is an efficient process for a wide range of cell types, irrespective of the host species, with any host range restriction occurring after viral entry [1]. The complex replication cycles of poxviruses take place exclusively in the cytoplasm, although it has long been suggested that poxviruses must interact with host nuclei for productive infection [2,3,4,5,6]. Perhaps the best-studied antiviral host restriction mechanism is interferon (IFN)-mediated, against which almost all viruses have evolved defence mechanisms [7,8]. Some of the first viral anti-IFN defence mechanisms were elucidated using vaccinia virus (VACV), which expresses multiple, often redundant inhibitors of IFN induction, JAK/STAT signalling and IFN-stimulated genes (ISGs), as well as IFN-receptor antagonists and mimics of IFN ligands [7,8,9,10,11,12].

These potent immunomodulators are produced mainly during the early phase of VACV gene expression. However, poxviruses have strategies in place to prevent or evade immediate–early host innate responses induced as a consequence of the virus binding and fusing with the cell membrane. Poxviruses have proteinaceous substructures, termed lateral bodies (LBs), outside the core but within the mature virion’s membrane. These are analogous to herpesvirus tegument proteins, some of which perform immunomodulatory functions early during infection [13,14,15,16].

Schmidt et al. [17] reported that VACV packages the conserved H1 phosphatase (also known as VH1) within LBs. When VH1 is released from LBs into the cytoplasm of the host cell following membrane fusion, it acts to block IFN-γ-mediated STAT-dependent signalling prior to gene expression [18]. Whether additional LB-resident viral immune modulators, capable of blocking other parts or the IFN system, are packaged in the LB of VACV or other poxviruses remain to be determined.

Relative to our understanding of the immunomodulation mediated by mammalian poxviruses, our knowledge of the strategies deployed by avipoxviruses to disarm the interferon response remains rudimentary. A prototypic member of the avipoxviruses, the fowlpox virus (FWPV) is the causal agent of a widespread, enzootic disease of domestic chickens and other gallinaceous birds [19]. Like VACV, it has been developed for use as a recombinant vector for the expression of antigens from several avian and human pathogens in both poultry and humans [19,20]. The commercial FWPV recombinant vaccine (TROVAC-H5) expressing the hemagglutinin gene of H5N8 isolate A/turkey/Ireland/1378/83 has become the most extensively used live recombinant virus used, with almost 2 billion doses used against highly pathogenic influenza H5N2 [20].

In common with the other poxviruses, FWPV has developed strategies to disarm the host IFN-response and has been found to efficiently block the pI:C-mediated induction of the IFN-β promoter and the IFN-stimulated induction of ISGs in chicken cells [20]. Studies of FWPV immunomodulators have been complicated by the fact that only 110 (42%) of FWPV genes share significant similarity to those in other poxviruses [21]. To identify the innate immunomodulatory factors encoded by FWPV, we previously conducted two broad-scale pan-genome analyses of FP9, a highly attenuated strain used as a vaccine vector in both poultry and mammals [21,22]. In the first study, we identified *FPV012* as a modulator of IFN induction by screening a knockout library of 65 nonessential FP9 genes [20,23]. In the second study, using a gain-of-function approach, in which 4–8 kbp fragments of FP9 were introduced into modified vaccinia Ankara (MVA), we found that FPV014 contributes to increased resistance to exogenous recombinant chicken IFN-α [24].

In this report, we use our existing FP9 knockout library [20] to screen infected primary chicken embryo fibroblasts (CEFs) for FWPV genes that modulate the induction of interferon-regulated genes (IRG)s. Using this approach, we identified FPV184 as a third FWPV immunomodulatory protein blocking the induction of innate immune responses. Intriguingly, unlike the FWPV immunomodulators FPV012 and FPV014 (which are both early viral proteins), FPV184 was found to be a late, structural protein with a functional nuclear localisation signal. Consistent with its ability to modulate ISG responses soon after infection and long before de novo production, we show that FPV184 is packaged into FWPV particles where it resides in LBs. These results suggest that the packaging of late immunomodulatory proteins and their subsequent delivery into the nucleus of newly infected cells serve as an immediate–early innate immune evasion strategy.

## 2. Experimental Section

### 2.1. Cells and Viruses

Freshly isolated chicken embryo fibroblasts (CEFs) were provided by the former Institute for Animal Health, Compton, Berks, UK (now the Pirbright Institute, UK). Primary CEFs derived from special SPF chicken lines are normally used for propagation and titration of FWPV [19]. CEFs were cultured in 199 media (Gibco^®^, Invitrogen, Carlsbad, CA, USA), supplemented with 8% heat-inactivated newborn calf serum (NBCS; Gibco^®^, Invitrogen), 10% tryptose phosphate broth (Sigma-Aldrich, St. Louis, MO, USA), 2% nystatin (Sigma-Aldrich) and 0.1% penicillin–streptomycin (Gibco^®^, Invitrogen). DF-1 is a CEF-derived, spontaneously immortalised cell line, which, unlike CEFs, exhibits high transfection efficiency and, at the same time, supports satisfactory propagation of FWPV [25]. HEK293T cells are immortalised human embryonic kidney cells that are commonly used in protein expression studies. DF-1 and HEK293T cells were maintained in Dulbecco modified Eagle medium (DMEM) supplemented with 10% fetal bovine serum (Gibco^®^, Invitrogen), and 0.1% penicillin–streptomycin. All cells were grown at 37 °C. The origin, propagation and titration (plaque assays) of the FP9 virus have been described previously [21,26,27]. All transient transfections in the study were conducted with Fugene HD transfection reagent (Promega, Southampton, UK) unless otherwise specified. To collect the purified virus, confluent CEFs were infected with FP9 at 0.1 PFU per cell. At 5 days p.i., the supernatant was harvested, along with remaining cells. Virus purification has been described previously [27].

### 2.2. Generation of Viable FPV184-Knockout Viruses (Deletion Mutant Viruses)

Generation of *FPV184* deletion mutant viruses was done using two different approaches—a PCR-mediated knockout with a guanine–phosphoribosyltransferase (GPT) insertion in the middle of the gene (FWPV∆184; used for the microarray study) and a transient dominant deletion mutant virus (TDdel184) [28]—to produce recombinant viruses. The resulting knockout viruses were sequenced in the region of *FPV184*, paying attention to the overlap with *FPV183*, to check that no adventitious mutations had been inadvertently introduced during the knockout procedure. The nearest flanking genes to *FPV184*, *FPV183* and *FPV186* are oriented such that the termini of the genes are towards *FPV184*, therefore making it unlikely that manipulation of *FPV184* would disrupt their promoters. Generation of dually tagged *FPV184* was not possible as the sequences encoding the C-termini of *FPV183* and *FPV184* overlap by (about) 30 bp.

#### 2.2.1. Isolation of FPV184 Deletion Mutant Virus (FWPV∆184) by PCR-Mediated Knockout

All primers used in the study are listed in Table S1. Insertional mutagenesis has been described previously [20,23]. PCR-mediated knockout of *FPV184* was carried out using three sets of primers; FWPV DNA was used as the template for PCR with primers M2840 to M2841 and M2844 to M2845, whilst the previously described vector pGPTNEB193rev [27] was used as the template for primers M2842 and M2843. The resultant PCR products were combined in equimolar amounts, and a further round of PCR was carried out using the flanking primer pair M2840 and M2845. The full-length PCR construct was then used to transfect FP9 infected-CEFs, and the recombinant viruses were selected using media containing mycophenolic acid (25 μg mL^−1^), xanthine (250 μg mL^−1^), and hypoxanthine (15 μg mL^−1^; MXH). The virus was passaged three times in T25 flasks in the presence of MXH before plaquing. The passaged virus was then plaque-purified once in the absence of MXH and once more in the presence of MXH.

#### 2.2.2. Isolation of FPV184 Deletion Mutant Virus (TDdel184) by Transient Dominant Selection

The *FPV184* deletion plasmid was constructed using the previously described vector pGNR [26]. The 5′ end of the *FPV184* gene and 200 bp of the upstream sequence were amplified by PCR using primer pair M2854 and M2856; the 3′ end of the *FPV184* gene and 200 bp of the downstream sequence were amplified using primer pair M2857 and M2855. Following this first round of PCR, the products were combined in equimolar amounts and a second round of PCR was carried out using the flanking primer pair M2854 and M2855. Utilising *BamH*I and *Hind*III sites within M2854 and M2855, respectively, the second round PCR product was digested and cloned into pGNR/*BamH*I/*Hind*III to produce pGNRFPVΔ184. Deletion mutant virus (TDdel184) was isolated by the transient dominant selection method [28], as described previously [26].

#### 2.2.3. Confirmation of FPV184 Knockout in Deletion Mutant Viruses (FWPV∆184 and TDdel184)

Each deletion mutant virus was screened by PCR with flanking primers (giving PCR products of specific sizes for wild-type and knocked-out genes), one flanking primer and one primer internal to the deleted sequences (detecting only the wild-type gene) or one flanking primer and one primer specific for the *GPT* gene (detecting the insertion of *GPT* into the wild-type gene). The primers used are as follows: flanking primers M530 TO M1257; internal primer M2919 to M1257; PCR-mediated deletion *GPT* primers M192 to M2854; transient dominant deletion *GPT* primers M192 to M1257.

#### 2.2.4. Generation of Recombinant EGFP-Expressing Viruses (EGFP wt, NLS^−^ and A19 NLS)

The *FPV184* gene was amplified by PCR with the primers M2952 and M2951. The product was digested with *Xma*I and *Sac*II (within M2952 and M2951, respectively) and cloned into the expression/transfer vector pEF*gpt*12S-CvectorEGFPmyc, which was derived from the vector pEF*gpt*12S [29,30,31] that was previously cloned with the coding sequence of EGFP (enhanced green fluorescent protein) from the pEGFP-C1 vector (Molecular Probes, Eugene, OR, USA) [29,31]. The cloning of the *FPV184* gene into pEF*gpt*12S-CvectorEGFPmyc produced pCVecEGFP184 (EGFP at the N-terminus). In addition, mutations were introduced into the *FPV184* gene by PCR using the following primer pairs: M2953 and M2951 for NLS^−^; M2956 and M2951 for VACV A19 NLS and two more controls to assess the effect of a putative phosphorylation site (PT; results not shown); primers M2954 and M2951 for NLS^−^/PT^−^; primers M2955 and M2951 for PT^−^. All of the products were digested with *Xma*I and *Sac*II and cloned into pEF*gpt*12S-CvectorEGFPmyc/*Xma*I/*Sac*II. Following transfection of constructs into FP9-infected CEF, recombinant EGFP-expressing viruses (EGFP wild-type (wt), NLS^−^ and A19 NLS) were selected using mycophenolic acid and plaque-purified twice.

### 2.3. Multistep Growth Curve

Confluent CEFs were infected with the PCR-mediated (FWPV∆184) and transient dominant (Tddel184) knockout viruses of FPV184 or with the wild-type virus (FP9) at 0.01 PFU per cell. The inoculum was removed 1 h later and replaced by fresh medium. At different times p.i., the extracellular medium was collected, and the cells were overlaid with 1 mL of fresh medium and stored at −70 °C. Intracellular and extracellular viruses were subjected to titer determination by plaque assay [32]. Plaque sizes between wild-type and knockout viruses were evaluated as before. Briefly, plaques areas were digitally enlarged and calculated in arbitrary units using ImageJ v.32 image analysis software. Scatterplots were created with GraphPad prism v.6.0 (GraphPad software. San Diego, CA, USA).

### 2.4. Generation of Expression Constructs (pcDNA6FPV184V5His, pFPV184, pVACV-A19)

The *FPV184* gene was amplified by PCR with M2892 and M2893. The product was digested with *Hind*III and *Xho*I and cloned into pcDNA6V5His/*Hind*III/*Xho*I (Invitrogen) to give pcDNA6FPV184V5His or amplified with 4279 and 4280 and digested with *XhoI* and *SalI* into pCIFLAG(N-terminus)/*XhoI*/*SalI* to give pFPV184. VACV A19 was amplified with 4344 and 4345 and digested with *XhoI* and *SalI* into pCIFLAG/*XhoI*/*SalI* to give pVACV-A19.

### 2.5. Infection of CEFs for Microarray and qPCR Analyses

Media was removed from fully confluent CEFs (in T25 flasks; Greiner Bio-one (Alphen a/d Rijn, The Netherlands); 5.6 × 10^6^ cells/flask) and replaced with 8 mL DMEM containing DMEM (mock-infected), FP9 (MOI; multiplicity of infection: 5) or one of the knockout viruses (MOI: 5). After 2 h, DMEM or virus-containing DMEM was replaced with culture media (199 media supplemented with 2% NBCS, 10% TPB, 2% nystatin and 0.1% penicillin/streptomycin) and cells were then incubated for a further 14 h before harvesting. Mock- and virus-infected cells were harvested at 16 h postinfection and stored at −80 °C in RNALater (Sigma-Aldrich) until RNA extraction. The experiment was repeated in triplicate for each knockout virus (for FWPVΔ228, virus duplicates were used) using three different batches of CEFs.

### 2.6. RNA Extraction and Processing of Samples for Microarray

RNA isolation and processing of samples and microarrays was done as previously described [25]. Total RNA was extracted from mock-, infected-, and IFN-stimulated DF-1 and CEFs using the RNeasy kit (Qiagen, West Sussex, UK) according to the manufacturer’s instructions, as previously described [25]. On-column DNA digestion was performed using RNase-free DNase (Qiagen) to remove contaminating genomic DNA. RNA samples were quantified using a Nanodrop spectrophotometer (Thermo Fisher Scientific, Waltham, MA, USA), and their quality was evaluated using a 2100 bioanalyser (Agilent Technologies, Santa Clara, CA, USA). All RNA samples had an RNA integrity number ≥9.6. RNA samples were processed for microarray using the GeneChip^®^ 3′ IVT Express Kit (Affymetrix, Emeryville, CA, USA) according to the manufacturer’s instructions, as previously described [25]. Total RNA (100 ng) was used as input, and quality checks were performed using the bioanalyser at all stages. RNA samples were processed in batches of 12, and batch mixing was used at every stage to avoid creating experimental bias. Hybridisation of RNA to chips and scanning of arrays was performed by the Medical Research Council’s Clinical Sciences Centre (CSC) Genomics Laboratory, Hammersmith Hospital, London, UK, as previously described. The RNA was hybridised to GeneChip Chicken Genome Array chips (Affymetrix; containing comprehensive coverage of 32,773 transcripts corresponding to over 28,000 chicken genes) in a GeneChip hybridisation oven (Affymetrix), the chips were stained and washed on a GeneChip Fluidics Station 450 (Affymetrix), and the arrays were scanned in a GeneChip Scanner 3000 7G with an autoloader (Affymetrix).

### 2.7. Microarray Data Analysis

A one-way ANOVA adjusted with the Benjamini–Hochberg multiple-testing correction (false discovery rate (FDR) of *p* < 0.05) was performed with Partek Genomics Suite (v6.6, Partek Inc, St Louis, MO, USA) across all samples, as previously described [25,33]. Comparisons were conducted between infected cells versus mock-treated cells and between infected cells with the KO viruses versus CEFs infected with the parental FP9 strain. The analysis cut-off criteria were fold change ≥ ±1.5 and *p*-value ≤0.05. The Affymetrix chicken genome arrays contain probe sets for detecting transcripts from 17 avian viruses, including FWPV, allowing confirmation of viral infection. Visualisation of gene expression data was conducted with GeneSpring GX (v.13.1, Agilent Technologies) and GraphPad Prism (v.6.0). Original microarray data produced in this study have been deposited according to the MIAME guidelines in the public database ArrayExpress (http://www.ebi.ac.uk/microarray-as/ae/; Acc. No: E-MTAB-7276). A catalogue of 337 ISGs was created by applying a fold change >3 compared to mock and false discovery rates (FDRs) <0.05 on previously published microarray data ([34]; ArrayExpress accession: E-MTAB-3711 and Table S2).

### 2.8. Quantitative Real-Time RT PCR

Quantitative real-time RT PCR was performed on RNA samples using a two-step procedure, as previously described [35]. RNA was first reverse-transcribed into cDNA using the QuantiTect Reverse Transcription Kit (Qiagen) according to manufacturer’s instructions. qPCR was then conducted on the cDNA in a 384-well plate with an ABI-7900HT Fast qPCR system (Applied Biosystems, Foster City, CA, USA). Mesa Green qPCR MasterMix (Eurogentec, Seraing, Belgium) was added to the cDNA (5 μL for every 2 μL of cDNA). The following amplification conditions were used: 95 °C for 5 min; 40 cycles of 95 °C for 15 s, 57 °C for 20 s, and 72 °C for 20 s; 95 °C for 15 s; 60 °C for 15 s; 95 °C for 15 s. Primer sequences for genes that were used in the study are shown in Table S1. The output Ct values and dissociation curves were analysed using SDS v2.3 and RQ Manager v1.2 (Applied Biosystems). Gene expression data were normalised against the housekeeping gene GAPDH and compared with the mock controls using the comparative C_T_ method (also referred to as the 2^−ΔΔCT^ method [36]). All samples were loaded in triplicate.

### 2.9. Confocal and Widefield Fluorescence Microscopy

Immunofluorescence labelling was carried out using CEFs seeded at 2.5 × 10^5^ cells/well on coverslips, incubated in 6-well plates at 37 °C, 5% CO_2_ and infected with 0.5–1 pfu of virus for 24 h. The medium was aspirated; cells were washed 3 times (3×) with PBS and fixed with 4% paraformaldehyde in PBS at room temperature (RT). Coverslips were washed 3× in PBS, and cells were permeabilised (0.5% Triton X-100 in PBS) at RT for 10 min with shaking. Following further 3× PBS washing, cells were blocked (0.5% bovine serum albumin in PBS) for 1 h at RT with shaking. Primary antibodies were applied at 1:200 in blocking solution for 1 h at RT with shaking, followed by 5× 5 min PBS washing at RT. Secondary Alexa dye-conjugated antibodies (Molecular Probes) were applied at 1:200 in blocking solution for 1 h at RT in the dark, followed by 5× 5 min PBS washing. For double-labelling experiments, a second round of incubation with primary antibody, washing and secondary antibody was carried out. To label DNA within cells, coverslips were incubated with 1:5000 TOPRO3 (Molecular Probes) for 10 min at RT, followed by 3× PBS washing. Coverslips were dipped in SuperQ water briefly, drained and mounted on Vectorshield mounting media (Vector Labs, Burlingame, CA, USA). Coverslips mounted with hard-set mounting media were allowed to set at 4 °C overnight; all other coverslips were sealed with nail varnish. Confocal microscopy was performed using a Leica TCS NT confocal microscope (Leica, Heidelberg, Germany).

For widefield fluorescence microscopy, CEFs were washed 2× with PBS at RT and fixed with 10% buffered formaldehyde. The images were acquired on Evos fluorescence microscope (Evos FL Imaging System, Thermo Fisher Scientific).

### 2.10. Virion Fractionation

Purified particles of the recombinant virus, which has EGFP fused with *FPV184,* were incubated in a reaction mixture containing 50 mM Tris-HCl, pH 7.5 and 1% (vol/vol) NP-40, with or without 50 mM dithiothreitol (DTT) for 1 h at 37 °C. The insoluble and soluble materials were separated by centrifugation at 20,000× *g* for 30 min at 4 °C.

### 2.11. Western Blotting

Proteins for Western blots were harvested from CEFs and DF-1 cells. Cell pellets were lysed with CelLytic-M solution (Sigma-Aldrich) and the supernatant collected by centrifugation at 15,000× *g*, 15 min, prior to protease inhibitor (Roche, Welwyn Garden City, UK) addition. To every 20 μL of sample, 5 μL of 4× loading buffer (Bio-Rad, Hemel Hempstead, UK) was added, and the samples were heated to 60 °C for 5 min. They were then separated on a 12% sodium dodecylsulfate polyacrylamide gel, alongside a protein ladder (Chameleon Dual Colour Standards, LICOR, Cambridge, UK). Samples (20 μg) were loaded for each well, and the gel was run at 150 V for 2 h. Protein samples fractionated by SDS-PAGE were electroblotted onto a nitrocellulose membrane (Hybond-ECL; Amersham Biosciences, Amersham, UK) by following standard protocols. After transfer, the membranes, blocked overnight in 5% nonfat dry milk in PBS buffer, were incubated for 2 h at room temperature with one of the following antibodies: mouse monoclonal anti-GFP (Sigma-Aldrich), mouse monoclonal anti-FLAG (M2) (Sigma-Aldrich), rabbit monoclonal anti-tubulin (Cell Signalling Technology; Ipswich, MA, USA), mouse polyclonal anti-chicken Mx1 (AbMart, Shanghai, China), and mouse monoclonal anti-FPV168 (GB9), anti-FPV140 (DF6) and anti-FPV191 (DE9; in PBS + 0.1% Tween-20 (Sigma-Aldrich) + 2% nonfat dried skimmed milk) [27,37] at a dilution of 1:(1000–5000), followed by washing with PBS five times for 5 min. Secondary antibody (goat anti-rabbit horseradish peroxidase-conjugated or goat anti-rabbit or donkey anti-mouse secondary antibodies (LICOR)) was diluted in PBS + 0.1% Tween-20 and added to the blot. Incubation was allowed to proceed for 1 h, followed by washing with PBS five times for 5 min each. Labelled proteins were detected by incubation with either the ECL detection reagent (Amersham Biosciences) and exposure to Hyperfilm ECL chemiluminescence film (Amersham Biosciences) or scanned with the Odyssey Imaging System (LICOR).

### 2.12. Transfection of Cells with POLYI:C and Assay of Luciferase Reporters 

Chicken fibroblast DF-1 cells were transfected in 12-well plates with either chicken Mx1 or chicken IFN-β promoter reporters (100 ng) [38], the constitutive reporter plasmid pJATlacZ (100 ng) [39] or cotransfected with plasmids driving the overexpression of FPV184 or FPV012 or VACV A19 or the empty control vector. Following recovery for 24 h, cells were either left untreated or treated with 1000 IU/mL recombinant IFN-α and incubated for 6 h or transfected overnight with high molecular weight polyI:C (10 μg/mL) purchased from InvivoGen (San Diego, CA, USA) using the Polyfect transfection reagent (Qiagen). Luciferase assays were carried out, and data were normalised using β-galactosidase measurements and expressed as relative luciferase activity. Cell lysate β-galactosidase concentrations were measured by incubation of 10 μL of cell lysate with ortho-nitrophenyl-β-galactoside (50 μL of 0.5 mg mL^−1^ diluted in 60 mM Na_2_HPO_4_·7H_2_O, 40 mM Na_2_H_2_PO_4_·H_2_O, 10 mM KCl, 1 mM MgSO_4_·7H_2_O, 2.7 mL litre^−1^ β-mercaptoethanol). The reaction mixture was incubated at 37 °C until a yellow colouration appeared; then, the *A*_420_ was measured using a spectrophotometer.

### 2.13. Correlative Super-Resolution Light and Electron Microscopy (CSRLEM)

Viruses expressing FPV184 with EGFP fused to its N-terminus were pelleted through 36% sucrose and then band-purified on a 25% to 40% sucrose gradient, as described previously [40]. Virions were diluted in 20 µl 1 mM Tris pH 9, placed in the centre of clean coverslips for 30 min and bound virus was fixed with 4% EM-grade formaldehyde (TAAB). A small asymmetric scratch was made in the middle of the coverslip using a diamond scorer to enable localisation of the super-resolution imaging region of interest within the resin block for trimming, targeting and, subsequently, in sections during electron imaging. The samples were permeabilised for 30 min with 1% Triton X-100 in PBS and blocked with PBS containing 5% BSA (Sigma-Aldrich), 1% FCS for 30 min. Then, the samples were immunostained overnight with anti-GFP nanobody (Chromotek, Martinsried, Germany), conjugated in-house to AlexaFluor647-NHS (Invitrogen) in PBS containing 5% BSA at 4 °C. Coverslips were washed 3 times with PBS and mounted on a microscope slide with parafilm gasket in 1% (*v*/*v*) β-mercaptoethanol (Sigma-Aldrich), 150 mM Tris, 1% glucose, 1% glycerol, 10 mM NaCl, pH 8 with 0.25 mg/mL glucose-oxidase and 20 µg/mL catalase.

Super-resolution microscopy was performed on an Elyra PS.1 inverted microscope (Zeiss, Dublin, CA, USA) using an alpha Plan-Apochromat 100×/1.46 NA oil DIC M27 objective. STORM [41] images were acquired with a 1.6× tube lens on an iXon 897 EMCCD camera (Andor) with 20 ms exposure time, 642 nm excitation at 100% laser power and a 655 nm LP filter. Fluorophore activation was dynamically controlled with a 405 nm laser at 0–2% laser power. Images were processed in Fiji [42] using ThunderSTORM [43]. Localisations were fitted with a maximum-likelihood estimator, and lateral drift was corrected by cross-correlation; localisations <20 nm apart within ≤1 frame were merged, and images were rendered using a Gaussian profile.

After super-resolution imaging of the region of interest, a series of phase-contrast images using objectives with 10×, 20×, 40× magnification and fluorescence images using an objective with 100× magnification were taken to map the region of interest and its localisation in relation to the scratch. Coverslips were washed twice in PBS and fixed with 1.5% glutaraldehyde and 2% EM-grade paraformaldehyde (TAAB) in 0.1 M sodium cacodylate for 45 min at room temperature. The samples were treated with reduced osmium and tannic acid, dehydrated through an ethanol series, and embedded in epon resin, as previously described [44]. After resin polymerisation, the coverslip was removed using liquid nitrogen, revealing the positive pattern of the coverslip-scratch on the surface of the resin block. Using the scratch mark as a reference and viral clusters from phase images as fiducials, the region of interest that had been imaged for super-resolution imaging was identified and targeted for serial sectioning. Sections were collected on formvar-coated slot grids, stained with lead citrate and imaged using a transmission electron microscope (Tecnai T12, Thermo Fisher Scientific, Waltham, MA, USA) equipped with a charge-coupled device camera (SIS Morada; Olympus, Tokyo, Japan). EM and STORM images were registered using NanoJ [45]. It should be noted that preparation of samples for EM after super-resolution microscopy may move or remove individual virus particles, which mandates careful registration and manual overlay of the images. However, the orientation and structure of the fluorescence signal are clearly reminiscent of the lateral body structure observed in EM.

### 2.14. Phylogenetic Analysis

The amino acid sequences of FPV184 orthologues from each genus of chordopoxvirus were subjected to multiple alignments using CLC Workbench 7 (CLC Bio, Qiagen, Aarhus, Denmark). Protein sequence accession numbers for the indicated viruses are as follows: fowlpox virus (FPV184, NP_039147), canarypox virus (CNPV258, NP_955281), VACV (A19, P68714), myxoma Virus (m109 L, AQT34599), deerpox virus (DpV83gp120, YP_227495), sheeppox virus (SPPV_106, NP_659683), swinepox virus (SPV108, NP_570268), Yaba monkey tumor virus (111L, NP_938366), molluscum contagiosum virus (MC124, AQY16697), orf virus (ORF096, NP_957873), and crocodilepox virus (P4b, YP_784314).

### 2.15. Statistical Analysis

To determine the significance of differences between experimental groups, a Shapiro–Wilk normality test was initially used to confirm whether the data followed a normal distribution for parametric or nonparametric testing. Subsequently, one- or two-way ANOVA parametric analyses were performed using the fold change scores with a Tukey’s or Dunnett’s multiple-comparisons test depending on the application [46]. *p*-values were set at 0.05 (*p* ≤ 0.05) unless indicated otherwise. Error bars represent the standard error of the mean (SEM). The correlation of expression values between microarray analysis and qRT-PCR was statistically assessed by calculation of Pearson’s correlation coefficient using the built-in function of GraphPad Prism (v.6.0).

## 3. Results

### 3.1. Identification of Immunomodulatory Signature Induced by FWPV∆184

We infected CEFs (three independent batches) with 59 individual FWPV knockout mutants, each deficient in one nonessential gene [20,23]. Gene expression was analysed at 16 h postinfection (p.i.) using Affymetrix Chicken 32 K genome microarrays, which include probe sets for FWPV transcripts, allowing confirmation of viral infection and genotype for each knockout virus. The parental FP9 strain blocks the induction of IFN and ISGs entirely and served as a negative control. The FPV012 knockout (FWPV∆012), which induces a subset of ISGs [20], served as a positive control. All knockout viruses were screened for their ability to induce ISGs using a set of 337 chicken ISGs, which were determined by treating CEFs with IFN-α for 6 h [34] (ArrayExpress accession: E-MTAB-3711; Table S2).

**Figure 1:**
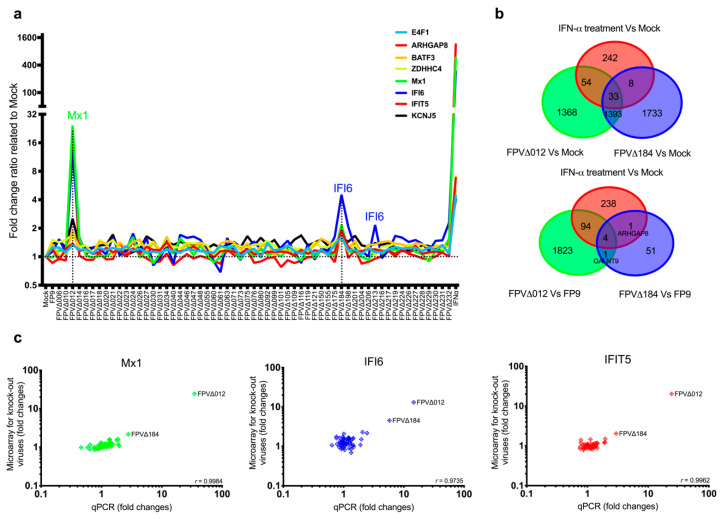
Disruption of *FPV012* (a known FP9 immunomodulator gene) and *FPV184*-induced overlapping subsets of IFN-stimulated genes (ISGs). (**a**) Gene expression of selected ISGs (E4F1, ARHGAP8, BATF3, ZDHHC4, Mx1, IFI6, IFIT5, KCNJ5Z) for all knockout viruses measured by microarrays (fold-change relative to mock-infected chicken embryo fibroblasts (CEFs)). (**b**) Venn diagrams showing numbers of genes differentially regulated by FWPV∆012, FWPV∆184 or IFN-α in comparison with mock-infected (upper) or parental FP9-infected (lower) cells. The full gene datasets are listed in Table S2. (**c**) Comparison of median differential expression levels for 3 selected transcripts determined by microarray and qPCR analysis (Mx1, IFI6, IFIT5). Plots show log10 expression fold change for the selected genes for CEFs infected with all knockout FP9 mutant viruses compared to mock-infected CEFs. Pearson correlation coefficients (*r*) are shown in the lower right corner of each plot.

FWPV∆012 induced a subset of ISGs to moderate levels compared to the mock-infected control (*n* = 87, FDR ≤ 0.05, fold change ≥1.5) and the FP9 virus (*n* = 98) (Figure 1a,b). We found that FWPV∆184 induced a smaller subset of ISGs compared with the mock-infected control (*n* = 41) and the FP9 virus (*n* = 5; IFIT5, Mx1, IFI6, ARHGAP8 (Rho GTPase activating protein 8), LOC418700 (LYG2 lysozyme G-like 2); Figure 1a,b and Table S2).

To confirm these results, we used real-time quantitative PCR (qPCR) to analyse mRNA levels of the “classical ISGs” Mx1, IFI6 and IFIT5 upon infection with the knockout viruses. Pearson’s correlation test (Figure 1c) was performed to test for pairwise correlations between the two transcriptomic approaches. The correlation coefficients (*r*) for all comparisons were over 0.97, indicating the reproducibility of the expression analysed by microarray or qPCR.

### 3.2. FPV184 Is Not an Essential Gene, but Its Loss Does Impart a Defect in Viral Growth

**Figure 2:**
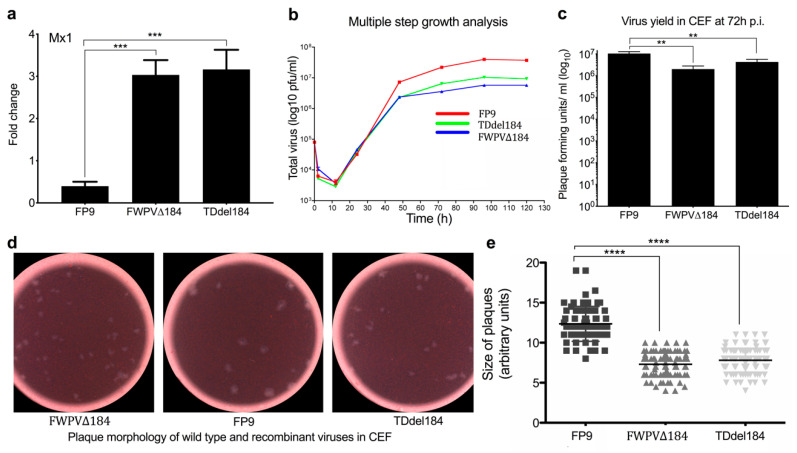
Deletion of *FPV184* affects the replicative fitness of the recombinant virus. (**a**) Independent transient dominant (TDdel184) and GPT-insertion *FPV184* knockout mutant (FWPV∆184) viruses (MOI (multiplicity of infection): 5, 1 h adsorption) induce similar mRNA levels of Mx1 at 4 h p.i. in CEFs compared to parental FP9 virus by qPCR. (**b**) Multiple-step growth analysis of FP9, FWPV∆184 and TDdel184 viruses in CEFs (MOI: 0.01). (**c**) Viral yield of FP9, FWPV∆184 and TDdel184 viruses (in pfu/mL) following 72 h infection with an MOI of 0.1 in CEFs. (**d**) Images of viral plaques of FP9, FWPV∆184 and TDdel184 viruses in CEFs. (**e**) Scatterplot showing their respective size in arbitrary units. (**a**–**e**) Error bars indicate standard error of the mean (SEM); *n* = 3. One-way ANOVA with Dunnett’s post hoc test was used to analyse the data. ** *p* < 0.01, *** *p* < 0.001, **** *p* < 0.0001.

FPV184 is a small protein (88 amino acids) of predicted molecular weight (9.5 kDa). The presence of a late promoter, TAAATG, upstream of *FPV184* suggested that it is a late-expressed gene unlike the other two known FWPV immunomodulators *FPV012* and *FPV014*, which are expressed early during viral replication. Microarrays showed that *FPV184* is indeed expressed at intermediate/late stages of FWPV replication (Figure S1c). To determine if *FPV184* is essential for FWPV replication, we used two recombinant FWPV viruses containing a disrupted *FPV184* gene, a PCR-mediated knockout virus (FWPV∆184; used in microarrays and the rest of the study) and a transient dominant virus (TDdel184). These deletion viruses could be isolated in culture, indicating that *FPV184* is nonessential for growth in vitro, and both are able to induce Mx1 in CEFs (Figure 2a). However, the viruses had lower growth rates (approx. 0.75 log_10_ lower), reduced viral yield in CEFs (approx. 1–1.5 log_10_ lower) and smaller plaque diameters (approx. two-thirds) compared to the parental FP9 virus (Figure 2b–e), indicating that loss of *FPV184* imparts a growth defect on FWPV.

### 3.3. FPV184 Mediates Early Suppression of ISGs through LB Packaging

**Figure 3:**
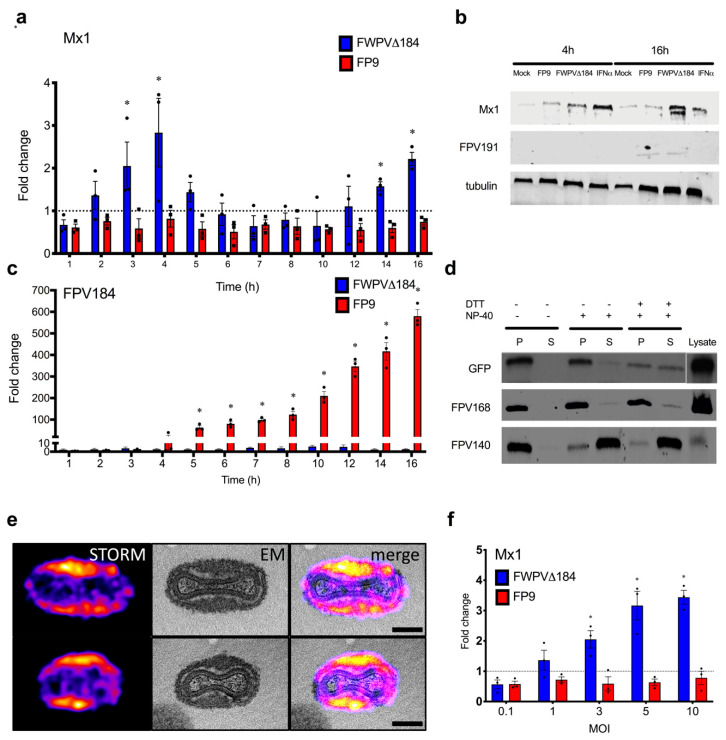
FPV184 mediates early suppression of ISGs through lateral body (LB) packaging. (**a**) Kinetics of Mx1 mRNA expression presented as scatter dot plots (means ± SEM, *n* = 3) following infection of CEFs with FWPV∆184 and the wild-type FP9 viruses (MOI: 5, 1 h adsorption), measured by qPCR. ● and ■ represent replicate values for infection with FWPV∆184 or FP9 respectively. (**b**) Western blot showing protein expression of chicken Mx1, late-expressed FPV191 and anti-tubulin at 4 and 16 h p.i. infection of CEFs with FP9 and FWPV∆184 (MOI: 5) and treatment with IFN-α (1000 IU/mL). (**c**) Kinetics of FPV184 expression presented as scatter dot plots (means ± SEM, *n* = 3) following infection of CEFs with FWPV∆184 and wild-type FP9 viruses, measured by qRT-PCR. (**d**) Purified particles of the virus, with EGFP fused with FPV184, were treated with either NP-40 and/or dithothreitol (DTT), centrifuged to separate the pellet (P) from supernatant (S) fractions, then subjected to SDS/PAGE and Western blotting. Immunodetection was performed with anti-GFP antibody to detect GFP fusion protein(s) and monoclonal antibodies against FWPV structural proteins FPV140 (localised on mature virions’ membrane) and FPV168 (localised in the virion core) [37]. (**e**) Correlative super-resolution light and electron microscopy of purified recombinant viruses, which have EGFP fused with *FPV184* and were immunolabelled with anti-GFP nanobody. STORM images of the lateral body protein were registered with electron microscopy (EM) micrographs. Two representative virions are shown at higher magnification in upper and lower panels (i and ii). Scale bars: 300 nm. An overview is shown in Figure S2. (**f**) mRNA expression levels of Mx1 in CEFs at 4 h p.i. infected with FP9 or FWPV∆184 using different MOIs (0.1, 1, 3, 5 and 10), measured by qPCR, and presented as scatter dot plots. (**a**,**c**,**f**) Columns represent fold-change (means ± SEM, *n* = 3) compared to mock-infected CEFs; two-way ANOVA with a Tukey’s posthoc test was used to analyse the data. * *p* < 0.05.

The expression kinetics of ISGs in CEFs infected with FWPV∆184 or FP9 were monitored for 16 h p.i. using qPCR. FP9 infection reduced basal Mx1 expression by 30% throughout the course of infection (Figure 3a shows mRNA expression of Mx1 infected with FP9 and FWPV∆184 related to uninfected samples). Conversely, cells infected with FWPV∆184 showed a modest bimodal increase in Mx1 expression from 2 to 5 h p.i. (2.8-fold compared with uninfected cells and 4.2-fold compared to FP9), and again at 14 to 16 h p.i. (2.2-fold compared with uninfected cells and 3.1-fold compared to FP9; Figure 3a). Immunoblot analysis at 4 and 16 h p.i. confirmed that FWPV∆184 could not suppress Mx1 protein expression (Figure 3b). As FPV184 is only expressed later during infection (Figure 3c and Figure S1), we confirmed late protein expression by immunoblot against another late protein (FPV191; Figure 3b), which confirmed that no late viral protein was expressed at 4 h p.i.

This late expression of FPV184 is unusual, if not currently unique, for a nonessential poxviral gene with early immunomodulatory function. The only known example of an immunomodulatory poxviral protein with an early effect is the essential VACV H1 phosphatase, which is packaged into poxvirus LBs and delivered into host cells during virus entry to mediate early suppression of STAT1 signalling [17]. Thus, we asked if FPV184 is packaged into FWPV virions and, if so, where in the virions it is located. Purified virions expressing FPV184 with EGFP fused to its N-terminus were left untreated or were subjected to fractionation using NP-40 and/or DTT to separate viral membranes from cores and their associated LBs [47]. Immunoblots directed against FWPV core protein FPV168 and membrane protein FPV140 were used to validate the fractionation [37]. Immunoblots directed against EGFP indicated that EGFP-fused FPV184 is packaged in virions (Figure 3d). In untreated and NP-40-treated virions, very little EGFP-fused FPV184 was released from virions, suggesting that the protein is not on the virions’ surface. Treatment with NP-40 and DTT [48] resulted in a 50/50 distribution of EGFP-fused FPV184 between virion membrane and core/LB fractions, suggesting that FPV184 resides between the viral membrane and the viral core.

To more accurately define the subviral localisation of FPV184, we used correlative super-resolution light and electron microscopy (CSRLEM). Purified virions expressing FPV184 with EGFP fused to its N-terminus were imaged using stochastic optical reconstruction microscopy (STORM), followed by transmission electron microscopy (TEM). Images were registered to correlate the fluorescence signal with EM structural information (Figure 3e and Figure S2). STORM images showed that FPV184 was localised to the two distinct LB structures running the length of the virions and absent from the virus cores. Correlation of these images with the corresponding TEM images showed that FPV184 is an LB resident protein. Consistent with its delivery via LBs, the activation of Mx1 gene expression at 4 h p.i. in cells infected with virions lacking FPV184 was found to be dose (MOI)-dependent (Figure 3f).

### 3.4. Ectopic Expression of FPV184 Inhibits polyI:C- and IFN-Stimulated Activation of Chicken IFN-β and Mx1 Promoters, Respectively

**Figure 4:**
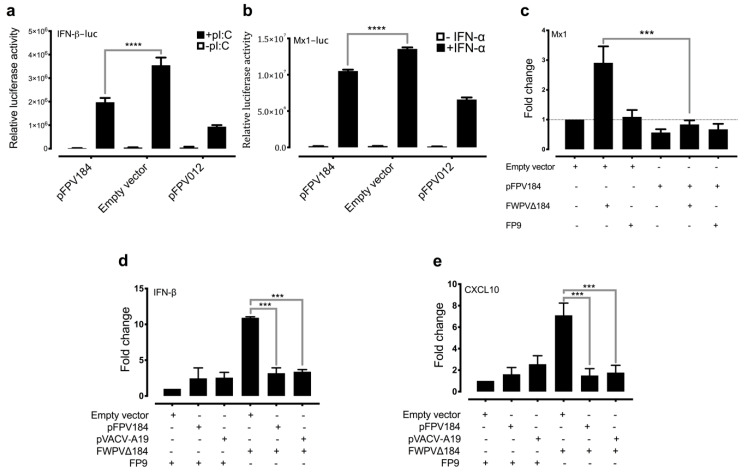
Ectopic expression of FPV184 elicits an immunomodulatory effect in chicken and human cells. Using a luciferase assay, the FLAG-tagged FPV184 protein was shown to inhibit the induction of both chicken IFN-β promoter (IFN-β-luc) by polyI:C (**a**) and, to a lesser extent, the chicken Mx1 promoter (Mx1-luc) by IFN-α (**b**) in chicken DF-1 cells. Data are presented as relative luciferase activity. Two-way ANOVA (type of plasmid and effect of IFN-α or pI:C) with Bonferroni’s posthoc test was used to analyse the data (**** *p* < 0.0001). (**c**) Complementation assay measuring Mx1 induction by qRT-PCR in DF-1 cells transiently transfected (48 h) with either the FPV184 expression plasmid or with the empty vector and infected with FWPV∆184 or FP9 for 4 h (MOI: 5). One-way ANOVA with Tukey’s posthoc test was used to analyse the data (*** *p* < 0.001). (**d**) qPCR analysis of human IFN-β and (**e**) human CXCL10 mRNA levels in HEK293T cells transfected for 48 h with either an expression plasmid for FPV184 or VACV A19 and infected with parental FP9 or FWPV∆184 for 4 h (MOI: 5). Error bars indicate SEM; *n* = 3. One-way ANOVA with Dunnett’s posthoc test was used to analyse the data. *** *p* < 0.001. (**c–e**) Results shown are representative of three independent experiments and show fold change in mRNA expression of genes compared to mock-transfected samples. Confirmation of transfections with qPCR for mRNA levels of FPV184 and VACV A19 is presented in Figure S5.

To directly assess the role of FPV184 in the absence of other immunomodulatory proteins expressed during infection, we used a construct (pFPV184) to overexpress FPV184 in immortalised DF-1 chicken fibroblast cells and assessed its ability to modulate the induction of the chicken IFN-β promoter by the dsRNA analogue polyI:C (Figure 4a) or the chicken Mx1 promoter by recombinant IFN-α, as detected using a luciferase reporter assay (Figure 4b). Compared to the empty vector, pFPV184 inhibited induction of the chicken IFN-β promoter and the Mx1 promoter by 44% and 22%, respectively. Overexpression of FPV012, which was used as a positive control for these experiments, inhibited induction by 74% and 51% for IFN-β and Mx1 promoters, respectively. Confirming its role in ISG suppression during infection, transient expression of FPV184 in DF-1 cells infected with FWPV∆184 resulted in wild-type ISG-suppression levels (Figure 4c). Cotransfection of the immortalised DF-1 chicken fibroblast cells with constructs expressing FPV184 and/or the other known FWPV immunomodulators, FPV012 and FPV014, showed that there is no synergism between the three proteins in inhibiting IFN-α stimulation of the Mx1 promoter (Figure S3).

### 3.5. Ectopic Expression of FPV184 and Its Ortholog, VACV A19, Restores the Immunomodulatory Capacity of FWPV∆184 in Human Cells

FPV184 is a well-conserved orthologue of VACV A19 (~40% amino acid identity; Figure S4), an essential, late structural protein found in vertebrate but not insect poxviruses [49,50]. As to whether VACV A19 could substitute FPV184, VACV A19 was overexpressed in human HEK293T cells infected with FWPV∆184 (Figure 4d,e and Figure S5). Under these conditions, expression of VACV A19 suppressed the induction of human IFN-β and CXCL10 as efficiently as FPV184, which served as a positive control for this experiment. The ability of A19 protein to compensate for the loss of FPV184 suggests that these proteins act as functional equivalents to suppress early immune responses upon infection.

### 3.6. FPV184 Contains a Functional NLS That Is Partially Responsible for Its Immunomodulatory Activity

**Figure 5:**
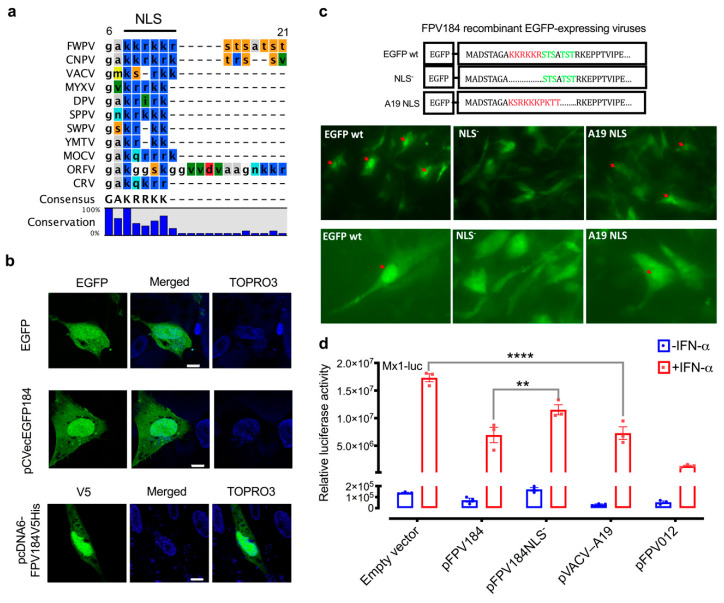
FPV184 NLS is functional, and nuclear localisation is needed for the protein’s immunomodulatory ability. (**a**) Multiple amino acid sequence alignments of FPV184 orthologues illustrate an NLS motif. Detailed information on sequences and full alignment can be found in the Materials and Methods section and Figure S4. (**b**) Representative confocal microscopy images of CEFs transfected for 48 h with expression plasmids containing either an EGFP tag alone (EGFP) or EGFP-tagged FPV184 (pCVecEGFP184) and infected with FP9 for 16 h or transfected for 48 h with an expression plasmid containing V5-tagged FPV184 (pcDNA6FPV184V5His) but left uninfected. Scale bar, 8 μm. (**c**) Upper panel: Schematic depicting modifications of the FPV184 N-terminal region in the recombinant EGFP-expressing viruses (NLS is shown in red; the putative phosphorylation sites in green). Lower panel: Widefield fluorescence micrographs of CEFs 24 h p.i. at an MOI of 1 with the recombinant EGFP-expressing viruses. Red arrows show nuclear-localised fluorescence. (**d**) Relative luciferase activity in DF-1 cells after transfection with chicken Mx1–luciferase reporter plasmid (Mx1–luc), together with expression plasmids for FPV184, FPV012, VACV A19 or FPV184 without the NLS domain (pFPV184NLS^−^). Data are presented as scatter dot plots. Two-way ANOVA with Tukey’s posthoc test was used to analyse the data (** *p* < 0.01, **** *p* < 0.0001). Error bars indicate SEM; *n* = 3.

It has been previously reported that VACV A19 and its orthologs, including FPV184, contain three highly conserved motifs: two CxxC motifs in the middle of the protein (amino acids 37–40 and 72–75), whose mutations resulted in a reduction in virus yield, and a basic NLS (FWPV amino acids 9–14: KKRKKR; Figure 5a and Figure S4) at its N-terminus [50]. While A19 was shown to display nucleo-cytoplasmic localisation during infection, mutation of the NLS had no apparent defect on virus growth [50].

To study the cellular localisation of FPV184 during infection, a construct expressing GFP/cMyc-tagged FPV184 under the control of an early/late synthetic poxvirus promoter was generated [31]. Consistent with the VACV A19 data, expression of this tagged version of FPV184 in FP9-infected CEFs showed its presence throughout the cytoplasm but was concentrated within the nucleus (Figure 5b). A nonfused EGFP control was fairly evenly distributed throughout the cytoplasm and nucleus. Expression of V5-His-tagged FPV184 from a eukaryotic expression vector, in the absence of infection, showed the same distribution (Figure 5b).

To determine whether the identified FPV184 NLS motif was responsible for this nuclear accumulation, we transiently expressed FPV184 lacking its NLS (Figure 5c; NLS^−^). To determine if the nuclear localisation signal found in FPV184 is functionally conserved with VACV A19, we also swapped this region of FPV184 with the corresponding sequence from VACV (strain Western Reserve (WR); NCBI Taxonomy ID 10254) A19 (KSRKKKPKTT) (Figure 5c; A19 NLS). These constructs were used to generate FWPV recombinant viruses expressing the EGFP-tagged versions of FWPV184 as second copies. Following infection with these viruses, EGFP wt showed diffuse cytoplasmic EGFP staining and distinct accumulation of the EGFP signal within both the viral factory and nucleus, while expression of the NLS^−^ recombinant EGFP virus produced a diffuse EGFP fluorescence in both nuclei and cytoplasms of infected cells (Figure 5c and confocal microscope analysis in Figure S6). Infection of cells with A19 NLS resulted in nuclear localisation, although the intensity was decreased compared to parental EGFP wt (Figure 5c and Figure S6). These results suggest that FWPV184 contains a functional NLS that is responsible for localising a steady-state portion of the viral protein into the cell nucleus throughout infection.

To assess the impact of the unusual FPV184 nuclear localisation on its ability to suppress Mx1, FPV184, FPV184 NLS- and FPV184 A19 NLS were transiently expressed in DF-1 cells and an Mx1–luciferase reporter assay was performed in the absence or presence of recombinant chicken IFN-α (Figure 5d). FPV184 partially suppressed IFN-mediated induction of the chicken Mx1 promoter, while FPV184 NLS- was reduced in its ability to suppress the promoter (Figure 5d). Consistent with its conserved sequence, VACV A19 NLS was able to rescue suppression by FPV184 A19 NLS to the level observed with FPV184 (Figure 5d).

## 4. Discussion

A common strategy to generate new or improved live recombinant poxvirus vaccines is to target and delete immunomodulatory genes in the vector. There has been considerable study of multiple immunomodulatory proteins of VACV [8,9,10,12] but, in contrast, few immunomodulators have been reported in other poxviruses. In FWPV, two immunomodulatory proteins have been reported so far: FPV012 and FPV014 [20,24]. Both are members of the ankyrin repeat protein superfamily (Pfam clan CLO465), are expressed early during viral replication and are not essential for virus replication in culture.

In a systematic, large-scale study, we interrogated the innate immune function of nonessential FWPV genes by transcriptomic analysis of a library of knockout viruses derived from the highly attenuated FWPV vaccine strain FP9. We identified a third immunomodulatory protein (FPV184), which, unlike the other two immunomodulators, is not an ankyrin protein, is expressed late during FP9 replication and encodes a very small structural protein packaged in the virions. There is a good degree of conservation between FPV184 and its VACV orthologue A19. A19 is also expressed at the intermediate/late stages of replication, but, unlike FPV184, it has been reported as essential for viral replication [50], being involved in the maturation of VACV virions and in early viral transcription in newly-infected cells, where it interacts directly with the viral RNA polymerase and other members of the early transcription complex [49,50]. Since A19 is a late protein and has an essential role in virion morphogenesis, A19 knockout viruses were not constructed and, thus, the potential role of A19 as an immunomodulator was not evaluated. Whilst conservation of A19 and FPV184 suggests an important function for the latter, we were able to generate FWPV lacking *FPV184* using two different methodologies, showing that the gene is nonessential for replication in vitro, albeit with a reduced replication rate.

Like VACV A19, FPV184 may act as an early viral transcription factor or a regulator. The two CxxC motifs involved in the binding of the VACV orthologue to viral RNA polymerase [49,50] are well conserved in FPV184. Although not investigated in this study, the presence of CxxC motifs is indicative of a zinc finger motif [51,52]. The zinc-binding domains, initially identified as DNA binding motifs in transcription factors, are now grouped into superfamilies based on their amino-acid composition. A number of transcription factors have been found to bind the DNA sequence (A/T)GATA(A/G) in the regulatory region of genes. These have been termed GATA-binding transcription factors and are able to bind DNA via a conserved zinc-finger domain in which the zinc ion is coordinated by four cysteine residues [53]. In VACV, early transcription factor null mutants have displayed defects in morphogenesis [50,54,55]. We have not conducted morphological studies of the FWPV∆184 virions, e.g., using electron microscopy; consequently, a role for FPV184 in morphogenesis cannot be excluded.

It is well established that FWPV blocks the launch of the avian type I IFN response and the induction of ISGs entirely. We recently showed that FWPV DNA is sensed by the chicken cGAS/STING DNA-sensing pathway, but the downstream signalling response leading to type I IFN production is effectively blocked by the wild-type virus [56]. Infection of chicken macrophages with FWPV∆184 resulted in IFN and ISG transcription, which was lost in cGAS and STING CRISPR knockout lines [56]. In this study, the relatively subtle induction of a subset of ISGs by FWPV∆184 could be explained by the presence of a multigenic, redundant system in FP9 to control host interferon response, as observed in VACV and other poxviruses or by relatively mild stimuli. It is possible that the complementary effects of other FWPV immunomodulatory proteins alleviate the effects caused by the absence of FPV184. For example, FPV138 is a homologue of VACV H1, a protein-tyrosine phosphatase that resides in the LBs, which, upon release into the cytoplasm, dampens type I IFN-induced, STAT1-mediated signalling. ISG expression was higher in cells infected with FWPV∆012 than with FPV∆184, in terms of both numbers and expression levels; there was, however, a significant overlap between the ISGs induced by both viruses (Figure 1b). Furthermore, when FPV012 and FPV184 were transiently coexpressed in DF-1 cells, they were not synergistic in inhibiting the pI:C-mediated induction of IFN-β (Figure S3), suggesting they may target the same host immune pathways but at different time points during infection. FPV184′s role may be to shut down immediate–early host immune responses and its function may be gradually superseded by that of the early immunomodulatory proteins expressed de novo upon infection.

The putative role of FPV184 in blocking immediate–early host immune response is also supported by our demonstration, with super-resolution microscopy and CSRLEM, that the protein is packaged within the LB and outside the confines of the viral cores, where early viral transcription is executed. LBs have been described as a poxvirus mechanism for the delivery of viral proteins to the cytoplasm of cells soon after fusion of the MV membrane with cellular plasma or endosomal membranes; their release and disaggregation are believed to depend upon reduction and proteasomal activity [17]. Parallels exist with the herpes virus tegument, which is also located inside the virion (under the envelope but outside of the capsid) and is known to deliver virion host effector proteins into cells [17,57]. Our hypothesis is that FWPV LBs contain additional packaged immunomodulatory factors that can act before core activation and early gene expression to establish a favourable environment in the cytoplasm. Other proteins found in LBs in VACV [17] include phosphoprotein F17 and oxidoreductase G4, both involved in morphogenesis, as well as the dual-specificity phosphatase H1 discussed above (FWPV orthologues, FPV103, FPV077 and FPV138, respectively). H1 has an immunomodulatory function by virtue of its ability to target STAT-mediated signalling, required for IFN-mediated induction of ISG expression. Our demonstration that FPV184 and VACV A19 are also packaged in LBs, with functional evidence that they both have an immunomodulatory function, shows that they could complement H1, primarily by blocking the induction of IFN but, to a lesser extent, also by blocking IFN-mediated induction of ISGs.

Another unexpected observation was that FPV184 is preferentially localised in the nuclei of host cells. Few host nuclear proteins have been shown to play a part in the poxvirus lifecycle, and fewer poxviral proteins (e.g., VACV C6, VACV F16) have been found to enter the nucleus during an infection [58,59]. There is no evidence that host proteins are required either for DNA replication or early gene transcription [60], but it is known that host proteins are necessary for VACV postreplication transcription; intermediate transcription requires host protein VITF-2, which resides within the nucleus of uninfected cells [61]. Although some poxviral proteins have been reported to enter the nucleus, to date, there has been no identification of a poxvirus-encoded protein containing an identifiable and functional NLS. Furthermore, the nuclear localisation of FPV184 was found to influence its immunomodulatory ability. Using a luciferase assay, we showed that a construct expressing FPV184 without the NLS only partially abrogated the ability of the protein to inhibit the IFN-mediated induction of the Mx1 promoter (Figure 5d). Whether nuclear localisation is partially or fully required for the immunomodulatory ability of FPV184 was unclear as, due to its small size (9.5 kDa), low levels of the protein can enter the nucleus without an NLS via passive diffusion. Myxoma virus encodes a protein termed myxoma nuclear factor (MNF) [62], an ankyrin repeat-containing protein that localises to the nucleus in the absence of an NLS and sequesters NF-κB. The cowpox virus protein CrmA, even though it is small enough to shuttle between the nucleus and the cytoplasm by passive diffusion, requires a leucine-rich nuclear export signal (NES) for its nuclear export [63]. It is possible that in the presence of the active NLS, the accumulation of FPV184 in the nucleus is dependent on its lack of an NES.

Although the conserved block of lysines and arginines found at the N terminus of FPV184 is characteristic of an NLS, it could also constitute a DNA/RNA binding domain. The possibility that FPV184 might be a dsRNA-binding protein could explain the inhibition of polyI:C mediated-induction of the IFN-β promoter but would not explain the inhibition of IFN-mediated induction of the Mx1 promoter we observed.

Collectively, the findings of this study indicate a late-expressed poxviral protein, packaged outside of the viral cores, with a nonessential phenotype, a functional NLS and an immediate–early effect on the innate immune response, properties that make FPV184 resemble a herpesvirus tegument protein rather than a typical early, immunomodulatory poxviral protein. The precise mechanism(s) and function(s) of FPV184 are obscure. Nevertheless, these findings extend the paradigm by which poxvirus structural proteins can block the induction of innate immune responses, immediately after infection, which might be useful in vaccine development.

## Data Availability

Microarray data are available at the public database ArrayExpress (http://www.ebi.ac.uk/microarray-as/ae/; Accession Number: E-MTAB-7276). All other data supporting the findings of this study are available from the corresponding author upon request.

## Supplementary Materials

The following are available online at https://www.mdpi.com/2227-9059/8/12/634/s1. Table S1: List of cloning, PCR and qPCR primers used in the study in the order they appear in the Materials and Methods section. Table S2: Excel workbook file (.XLS) with 6 spreadsheets showing full lists of the upregulated genes, which were determined in the 6 comparisons, as summarised in Figure 1b. Each individual tab shows one of the following comparisons: (a) FP9-infected versus mock-infected CEFs, (b) FWPV∆012-infected versus mock-infected CEFs, (c) FWPV∆184-infected versus mock-infected cells, (d) IFN-α-stimulated versus mock-stimulated CEFs, (e) FWPV∆012-infected versus FP9-infected CEFs, and (f) FWPV∆184-infected versus FP9-infected CEFs. The headings are Probeset ID, Gene Symbol, Gene Title, RefSeq Transcript ID, *p*-value (FDR) and Fold-change. Figure S1: (a) Log2 expression levels of FPV184 (chicken array probe: NC-002188.CDS185.S1_at) in mock-infected and infected CEFs with FP9 and knockout viruses. (b) Log2 expression levels of FPV094 (chicken array probe: NC-002188.CDS95.S1_at) in mock-infected and infected CEFs with FP9 and knockout viruses. (c) Intermediate/late transcription of *FPV184* is consistent for a structural gene but unusual for an immunomodulatory gene, which is usually expressed at early stages of infection. The figure shows log2 expression values determined by microarrays of *FPV184* and *FPV168* (late; structural genes), as well as *FPV012* and *FPV014* (early; immunomodulatory genes) during infection of CEFs with FP9 (at 4, 8, 16 and 24 h p.i.). Analysis of microarray data (ArrayExpress accession: E-MTAB-5455) was conducted with Genespring GX (Agilent). Figure S2: Correlative super-resolution light and electron microscopy of virions expressing FPV184 fused with EGFP at its N-terminus, immunolabelled with anti-GFP nanobodies. STORM images of the FPV184 protein were registered with EM micrographs of whole virions. Overview of two representative virions (i and ii), shown in higher magnification in Figure 3e. Scale bar: 2 µm. Figure S3: Luciferase reporter gene assay. DF-1 cells were transfected for 48 h with *FPV012*, *FPV014* and/or *FPV184* expression plasmids and a luciferase reporter plasmid directed by the chicken Mx1 promoter, as well as an internal control. Cells were either treated overnight with chicken recombinant IFN-α (1000 IU/mL) or left untreated. “Uninduced” corresponds to cells transfected only with the reporter plasmid. The relative luciferase activities are shown. Figure S4: Multiple amino acid sequence alignments of FPV184 orthologues from each genus of chordopoxvirus: CNPV (canarypox virus), VACV (vaccinia virus), MYXV (myxoma virus), DPV (deerpox virus), SPPV (sheeppox virus), SWPV (swinepox virus), YMTV (Yaba monkey tumour virus), MOCV (molluscum contagiosum virus), ORFV (Orf virus), and CRV (crocodilepox virus). The alignment was performed by importing the corresponding amino acid sequences into CLC Workbench (CLC Bio, Qiagen, Aarhus, Denmark). Figure S5: qPCR analysis of FPV184 or VACV A19 confirming transfections presented in Figure 4d,e. HEK293T cells were infected with parental FP9 or FWPV∆184 for 4 h (MOI: 5) and/or transfected with FPV184 or VACV A19 expression plasmids. Error bars indicate SEM; *n* = 3. One-way ANOVA with Dunnett’s posthoc test was used to compare the induction of mRNA expression against that of cells transfected with the empty vector. **** *p* < 0.001. Figure S6: Confocal analysis of CEFs infected with recombinant EGFP-expressing viruses, as summarised in Figure 5c. Confocal microscopy was performed using a Leica TCS NT confocal microscope.
