# Supplementary material for: Modulation of Early Host Innate Immune Response by an Avipox Vaccine Virus’ Lateral Body Protein
Source: Biomedicines. 2020 Dec 19;8(12):634. doi: 10.3390/biomedicines8120634 (PMC7766033; doi:10.3390/biomedicines8120634)

Modulation of early host innate immune response by an avipox vaccine virus' lateral body protein

Efstathios S Giotis[1,2*], Stephen M Laidlaw[1], Susanna R Bidgood[3], David Albrecht[3], Jemima J Burden[3], Rebecca C Robey[1], Jason Mercer[3] and Michael A Skinner[1]

1. Section of Virology, School of Medicine, St Mary's Campus, Imperial College London W2 1PG, UK.
2. School of Life Sciences, University of Essex, Colchester C04 3SQ, UK.
3. Medical Research Council-Laboratory for Molecular Cell Biology, University College London, Gower Street, London WC1E 6BT, UK.

*Correspondence to: e.giotis@imperial.ac.uk

Supplementary Materials:

Supplementary Tables

Table S1: List of cloning, PCR and qPCR primers used in the study, in the order they appear in the Materials and Methods.

Table S2: Excel workbook file (.XLS) with 6 spreadsheets showing full lists of the upregulated genes, which were determined in the 6 comparisons as summarised in Fig. 1b. Each individual tab shows one of the following comparisons: **(a)** FP9-infected versus mock-infected CEF, **(b)** FWPVΔ012 -infected versus mock-infected CEF, **(c)** FWPVΔ184 -infected versus mock-infected cells, **(d)** IFN-α stimulated versus mock-stimulated CEF, **(e)** FWPVΔ012 -infected versus FP9-infected CEF, and **(f)** FWPVΔ184 -infected versus FP9-infected CEF**.** Headings are: Probeset ID, Gene Symbol, Gene Title, RefSeq Transcript ID, p-value (FDR) and Fold-change.

Supplementary figures

Figure S1: **(a)** Log2 expression levels of FPV184 (Chicken array probe: NC-002188.CDS185.S1_at) in mock-infected, and infected CEF with FP9 and knock-out viruses. **(b)** Log2 expression levels of FPV094 (Chicken array probe: NC-002188.CDS95.S1_at) in mock-infected, and infected CEF with FP9 and knock-out viruses. **(c)** Intermediate/late transcription of *FPV184* is consistent for a structural gene but unusual for an immunomodulatory gene, usually expressed at early stages of infection. Figure shows log2 expression values determined by microarrays of: *FPV184* and *FPV168* (late; structural genes) as well as *FPV012* and *FPV014* (early; immunomodulatory genes) during infection of CEF with FP9 (at 4, 8, 16 and 24 h p.i.). Analysis of microarray data (ArrayExpress accession: E-MTAB-5455) was conducted with Genespring GX (Agilent).

Figure S2: Correlative super-resolution light and electron microscopy of virions expressing FPV184 fused with EGFP at its N-terminus, immunolabelled with anti-GFP nanobodies. STORM images of the FPV184 protein were registered with EM micrographs of whole virions. Overview of two representative virions (i and ii), which are shown in higher magnification in Fig. 3e. Scale bar: 2 µm.

Figure S3: Luciferase reporter gene assay. DF-1 cells were transfected for 48 h with *FPV012*, *FPV014* and/or *FPV184* expression plasmids and a luciferase reporter plasmid directed by the chicken Mx1 promoter, as well as an internal control. Cells were either treated overnight with chicken recombinant IFN-α (1000 IU/ml) or left untreated. 'Uninduced' correspond to cells transfected only with the reporter plasmid. The relative luciferase activities are shown.

Figure S4: Multiple amino acid sequence alignment of FPV184 orthologues from each genera of chordopoxviruses: genus of the chordopoxviruses: CNPV (canarypox virus), VACV (vaccinia virus), MYXV (myxoma virus), DPV (deerpox virus), SPPV (sheeppox Virus), SWPV (swinepox virus), YMTV (Yaba monkey tumor virus), MOCV (molluscum contagiosum virus), ORFV (Orf virus), CRV (crocodilepox virus). The alignment was performed by importing the corresponding amino acid sequences into CLC Workbench (CLC Bio, Qiagen, Aarhus, Denmark).

Figure S5: qPCR analysis of FPV184 or VACV A19 confirming transfections presented in Fig. 4d-e. HEK293T cells were infected with parental FP9 or FWPVΔ184 for 4 h (MOI: 5) and/or transfected with FPV184 or VACV A19 expression plasmids. Error bars indicate SEM; n=3. One-way ANOVA with Dunnett's *post hoc* test were used to compare induction of mRNA expression against that of cells transfected with the empty vector. ****p<0.001.

Figure S6: Confocal analysis of CEF infected with recombinant EGFP-expressing viruses as summarised in Fig. 5c. Confocal microscopy was performed using a Leica TCS NT confocal microscope.

S1 Table

Name	Sequence (5'-3')	Role
**M2840**	GCTTCTAAATTTATCAAACATC	PCR cloning to create FPVΔ184 with insertional mutagenesis
**M2841**	ATCGAGAACACAACTTTGACTGGCATATAG	PCR cloning to create FPVΔ184 with insertional mutagenesis
**M2842**	GTCAAAGTTGTGTTCTCGATGTCGAATAGC	PCR cloning to create FPVΔ184 with insertional mutagenesis
**M2843**	GAAAACATAACGGATGCAGCCTCCAGAATAC	PCR cloning to create FPVΔ1844 with insertional mutagenesis
**M2844**	GGCTGCATCCGTTATGTTTTCTGGGGTTAG	PCR cloning to create FPVΔ184 with insertional mutagenesis
**M2845**	CATCGATAAAGGAAATAAAATC	PCR cloning to create FPVΔ184 with insertional mutagenesis
**M2854**	CGCGGATCCCTTGATAAATTATAGCTTTACACC	PCR cloning to create transient dominant FPVΔ184
**M2856**	GATCCGCACGTCGTGTGGATGTAGCGGATGTAGATC	PCR cloning to create transient dominant FPVΔ184
**M2857**	CATCCACACGACGTGCGGATCTTCATTATGCC	PCR cloning to create transient dominant FPVΔ184
**M2855**	CGCGCGAAGCTTATATCCCAAACTTAGATAGCCTGTTC	PCR cloning to create transient dominant FPVΔ184
**M530**	CACCGTTACAAAAAATCC	PCR primer to confirm FPVΔ184 (flanking)
**M1257**	AGGCAAATATACGGAAAC	PCR primer to confirm FPVΔ184
**M2952**	GCGCCCGCGGGCGCAGACTCTACAGCAGGTGCTAAG	PCR cloning of FPV184
**M2951**	CGCGCCCGGGTTAGGATTTAGAGAGGTCTCTCAAATGGC	PCR cloning of FPV184 and GFP184 mutant viruses
**M2953**	GCGCCCGCGGGCGCAGACTCTACAGCAGGTGCTTCTACATCCG CTACATCCACACG	PCR cloning of GFP184Mut1
**M2954**	GCGCCCGCGGGCGCAGACTCTACAGCAGGTGCTGCTGCTGCTG CTCGTAAAGAACCGCCTACCGTTATACC	PCR cloning of GFP184Mut2
**M2955**	GCGCCCGCGGGCGCAGACTCTACAGCAGGTGCTAAGAAGAGG AAGAAAAGAGCTGCTGCTGCTACATCCACACGTAAAGAACCG CCTACCG	PCR cloning of GFP184Mut3
**M2956**	GCGCCCGCGGGCGCAGACTCTACAGCAGGTGCTAAGAGCCGC AAAAAGAAGCCCAAGACTACCGTTATACCAGAAGATGAA	PCR cloning of GFP184Mut4
**M2892**	CGCGAAGCTTATTATGGCAGACTCTACAGCAGGTGC	PCR cloning of FPV184V5
**M2893**	CGCGCTCGAGGGATTTAGAGAGGTCTCTCAAATG	PCR cloning of FPV184V5
**M4279**	GATCCTCGAGGCAGACTCTACAGCAGGTGCTAAG	FPV184 forward primer to clone into pCI-FLAG/Xho/Sal
**M4280**	GATCGTCGACTTAGGATTTAGAGAGGTCTCTCAAATG	FPV184 reverse primer to clone into pCI-FLAG/Xho/Sal
**M4344**	GATCCTCGAGGATAGCACCAATGTGCGTTCCGGAATG	Forward primer for cloning of VV A19 in pCI+FLAG-Xho site
**M4345**	GATCGTCGACTTAAATAGAATATTTAACTCGCAAAATC	Reverse primer for cloning of VV A19 in pCI+FLAG-SAL site
**chGAPDH**	GGCACTGTCAAGGCTGAGAA	qRT-PCR forward primer
**chGAPDH**	TGCATCTGCCCATTTGATGT	qRT-PCR reverse primer
**chMx1**	CACACCCAACTGTCAGCGAT	qRT-PCR forward primer
**chMx1**	ATGTCCGAAACTCTCTGCGG	qRT-PCR reverse primer
**chIFI6**	TGACCAGAACGTCCACAAAGCCG	qRT-PCR forward primer
**chIFI6**	ACCTGCTCCTGGACCGATGCTT	qRT-PCR reverse primer
**chIFIT5**	TGCTTCACCAGCTAGGACTCTGC	qRT-PCR forward primer
**chIFIT5**	TGGCTTTTGCTCTGTCACCACTTTG	qRT-PCR reverse primer
**FPV168**	ACCTCAAACAACCTCATC	qRT-PCR forward primer
**FPV168**	GTTAATACTTGTGACTGCTG	qRT-PCR reverse primer
**M2919**	CCTGTATTTAGATAATCGC	PCR primer to confirm FPVΔ184 (internal)
**M192**	AGGTGACGATGTATTTTTCGCTCAT	PCR primer to confirm FPVΔ184 (GPT)
**M2854**	CGCGGATCCCTTGATAAATTATAGCTTTACACC	PCR primer to confirm FPVΔ184 (GPT)

S1 Figure

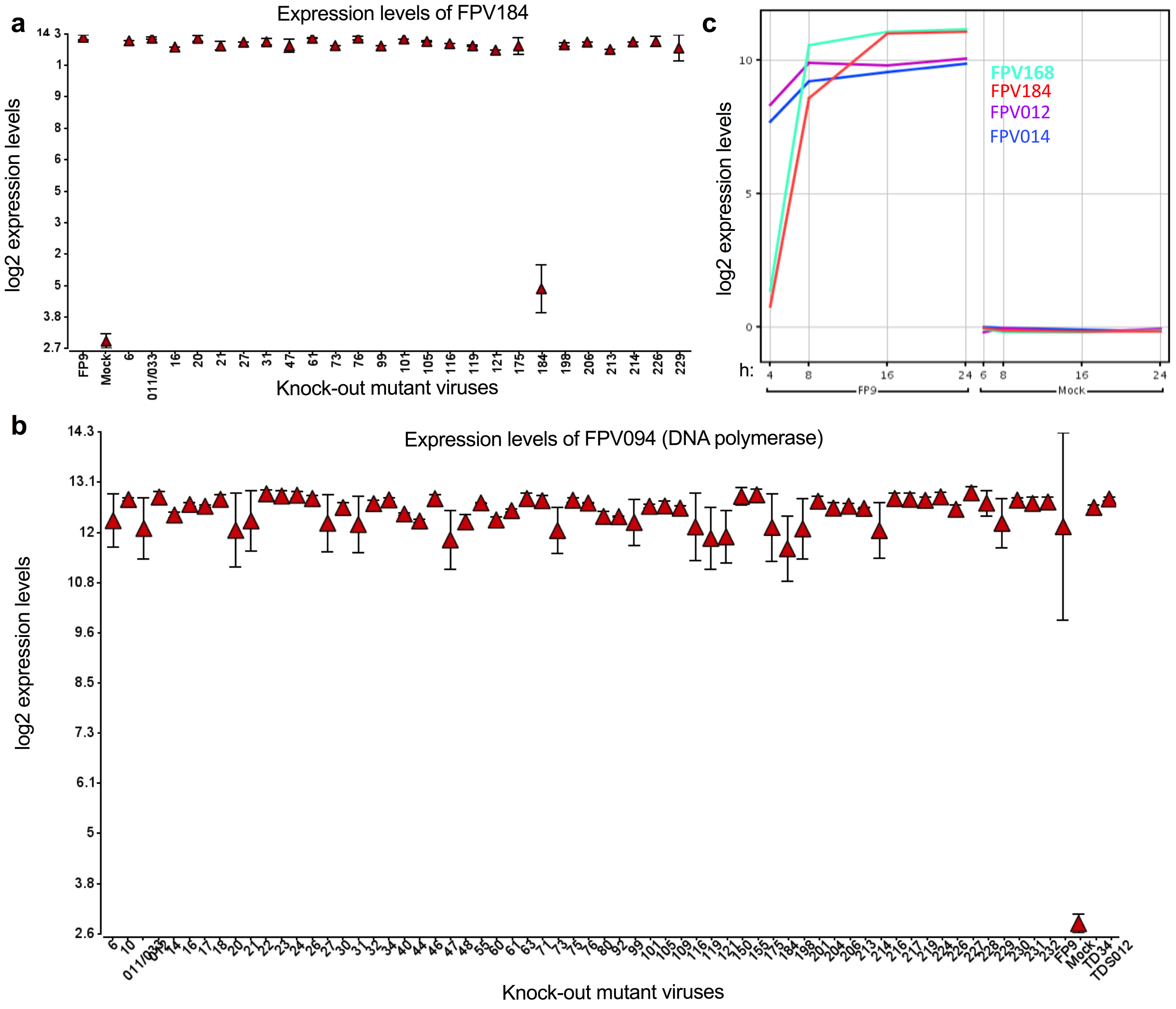
a
Expression levels of FPV184
log2 expression levels
Knock-out mutant viruses
c
log2 expression levels
FPV168
FPV184
FPV012
FPV014
h:
FP9
Mock
b
Expression levels of FPV094 (DNA polymerase)
log2 expression levels
Knock-out mutant viruses

S2 Figure

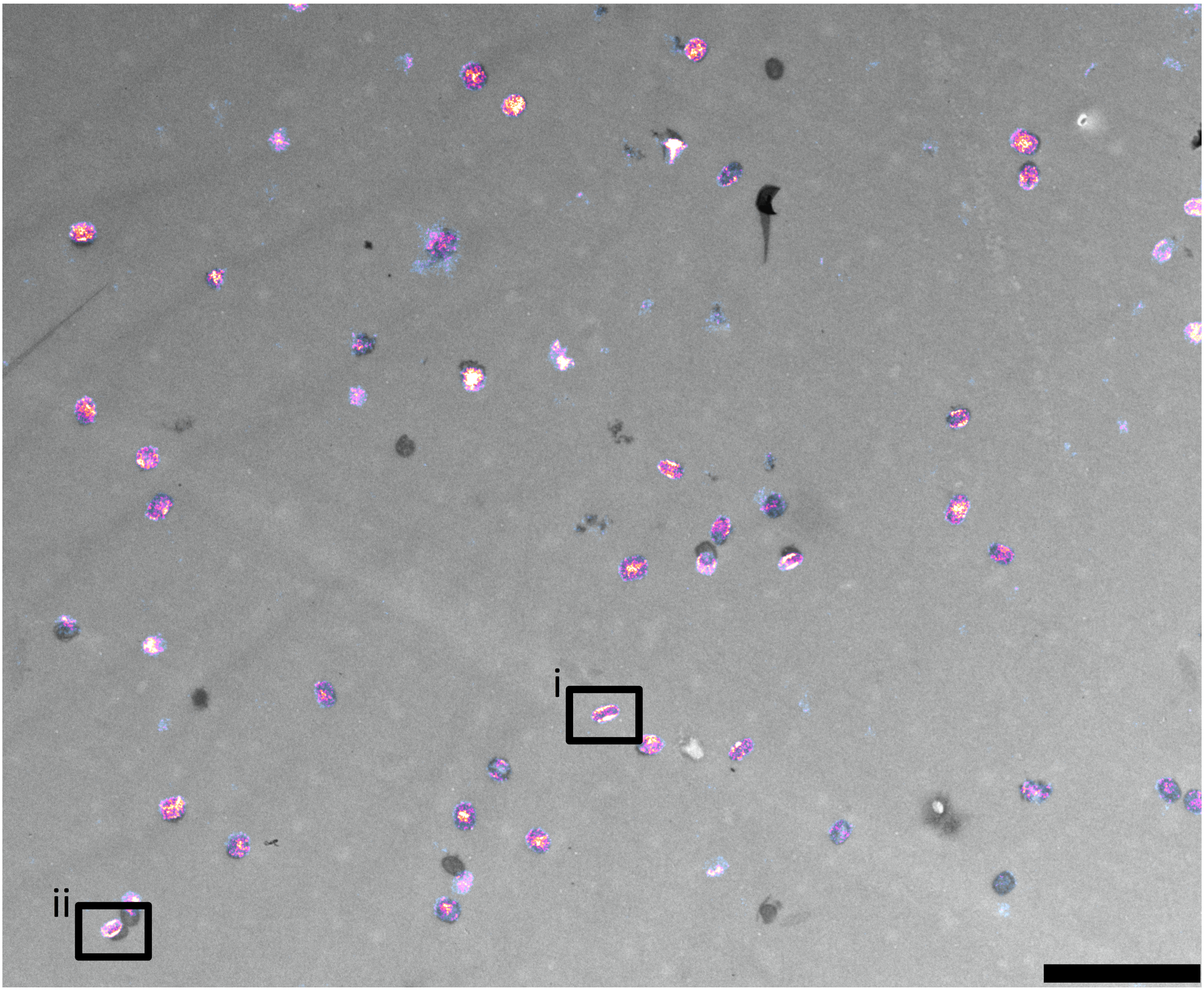

S3 Figure

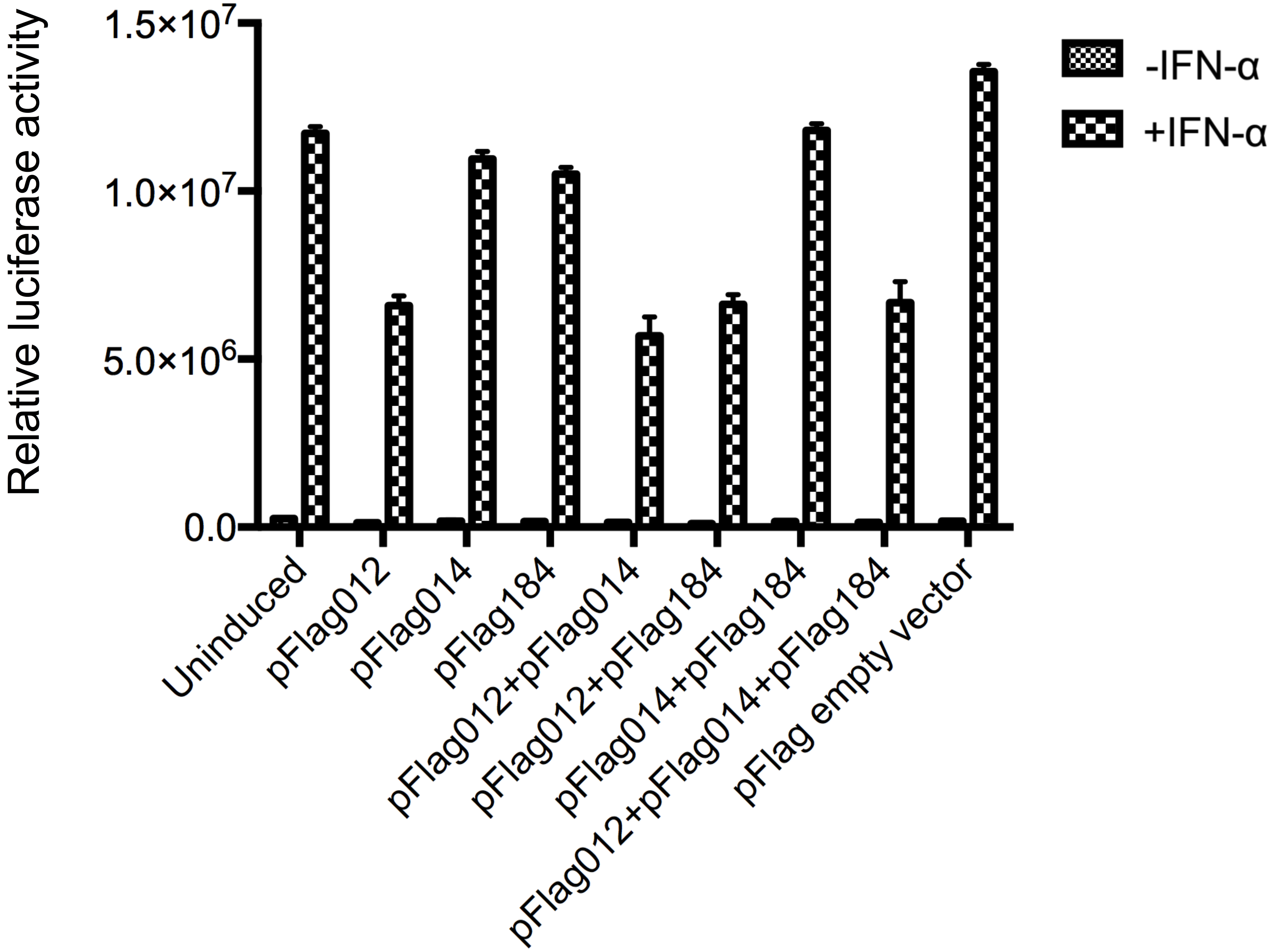
Relative luciferase activity
1.5×10^7
1.0×10^7
5.0×10^6
0.0
-IFN-α
+IFN-α
Uninduced
pFlag012
pFlag014
pFlag184
pFlag012+pFlag014
pFlag012+pFlag184
pFlag014+pFlag184
pFlag012+pFlag014+pFlag184
pFlag empty vector

S4 Figure

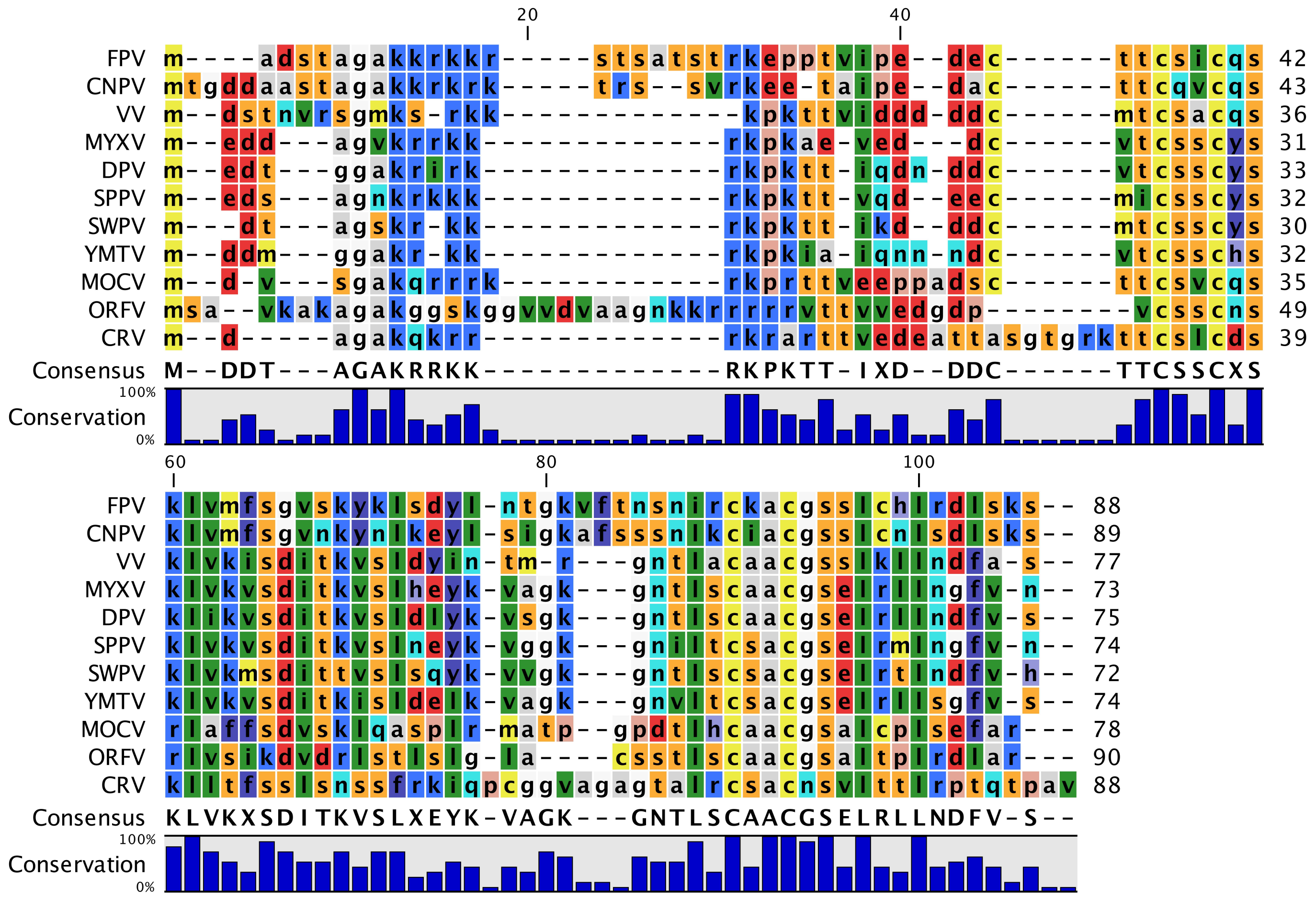
20
40
FPV m----adstagakkrkkr-----ststatstrkepptvipe--dec------ttcsicqs 42
CNPV mtgddaastagakkrkrk-----trs--svrkee-taipe--dac------ttcqvcqs 43
VV m--dstnvrsgmks-rkk-------------kpkttviddd-ddc------mtcsacqs 36
MYXV m--edd---agvkrrkk--------------rkpkae-ved---dc------vtcsscys 31
DPV m--edt---ggakrirk--------------rkpktt-iqdn-ddc------vtcsscys 33
SPPV m--eds---agnkrkkk--------------rkpktt-vqd--eec------micsscys 32
SWPV m---dt---agskr-kk--------------rkpktt-ikd--ddc------mtcsscys 30
YMTV m--ddm---ggakr-kk--------------rkpkia-iqnn-ndc------vtcsschs 32
MOCV m--d-v---sgakqrrrk-------------rkprttveeppadsc------ttcsvcqs 35
ORFV msa--vkakagakggskggvvdvaagnkkrrrrrvttvvedgdp--------vcsscns 49
CRV m--d-----agakqkrr--------------rkrarttvedeattasgtgrkttcslcds 39
Consensus M--DDT---AGAKRRKK-------------RKPKTT-IXD--DDC------TTCSSCXS
100%
Conservation
0%
60
80
100
FPV klvmfsgvskyklsdyl-ntgkvftnsnirckacgsslchlrdlsks-- 88
CNPV klvmfsgvnkynlkeyl-sigkafsssnlkciacgsslcnlsdlsks-- 89
VV klvkisditkvsldyin-tm-r---gntlacaacgsslkllndfa-s-- 77
MYXV klvkvsditkvslheyk-vagk---gntlscaacgselrllngfv-n-- 73
DPV klikvsditkvsldlyk-vsgk---gntlscaacgselrllndfv-s-- 75
SPPV klvkvsditkvslneyk-vggk---gniltcsacgselrmlngfv-n-- 74
SWPV klvkmsdittvslsqyk-vvgk---gntlscsacgselrtlndfv-h-- 72
YMTV klvkvsditkislde lk-vagk---gnvltcsacgselrllsgfv-s-- 74
MOCV rlaffsdvsklqasplr-matp--gpdtlhcaacgsalcplsefar--- 78
ORFV rlvsikdvdrlstlslg-la----csstlscaacgsaltplrdlar--- 90
CRV klltfsslsnssfrkiqpcggvagagtalrcsacnsvlttlrptqtpav 88
Consensus KLVKXSDITKVSLXEYK-VAGK---GNTLSCAACGSELRLLNDFV-S--
100%
Conservation
0%

S5 Figure

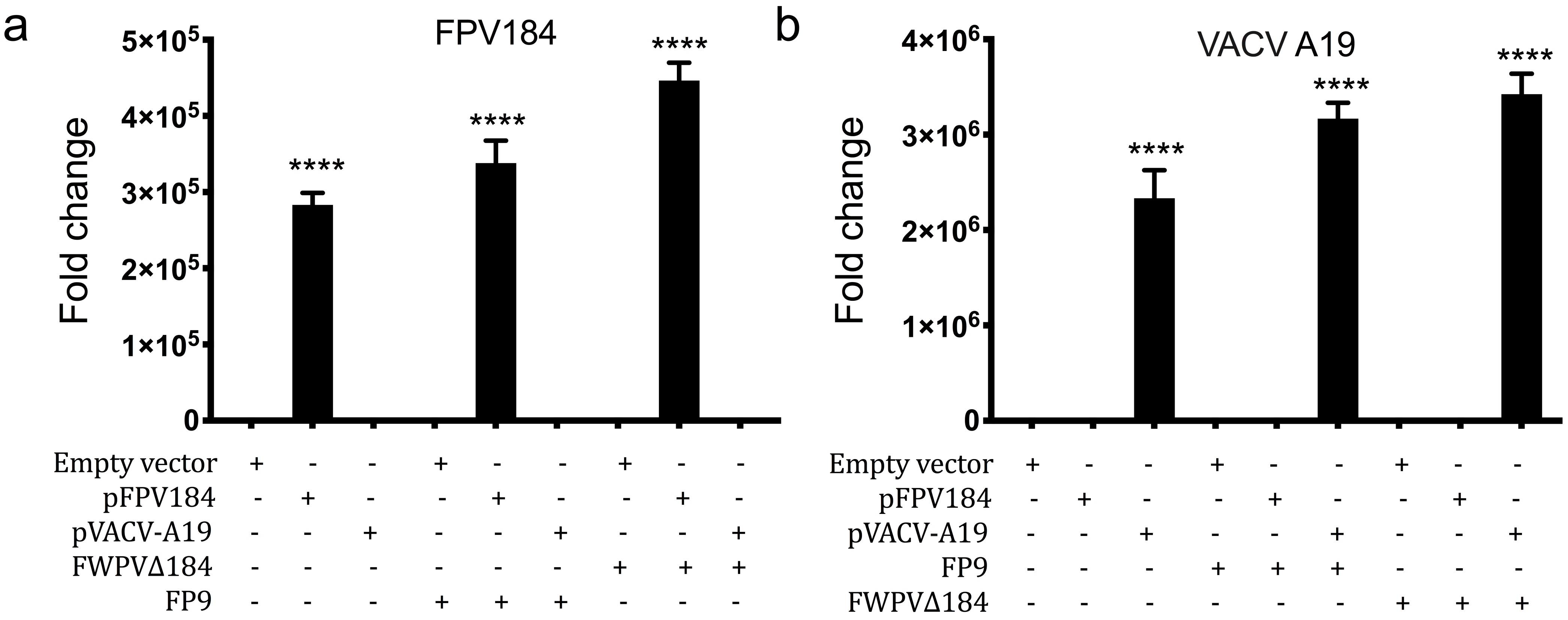
a
FPV184
Fold change
5×10^5
4×10^5
3×10^5
2×10^5
1×10^5
0
****
****
****
Empty vector + - - + - - + - -
pFPV184 - + - - + - - + -
pVACV-A19 - - + - - + - - +
FWPVΔ184 - - - - - - + + +
FP9 - - - + + + - - -
b
VACV A19
Fold change
4×10^6
3×10^6
2×10^6
1×10^6
0
****
****
****
Empty vector + - - + - - + - -
pFPV184 - + - - + - - + -
pVACV-A19 - - + - - + - - +
FP9 - - - + + + - - -
FWPVΔ184 - - - - - - + + +

S6 Figure

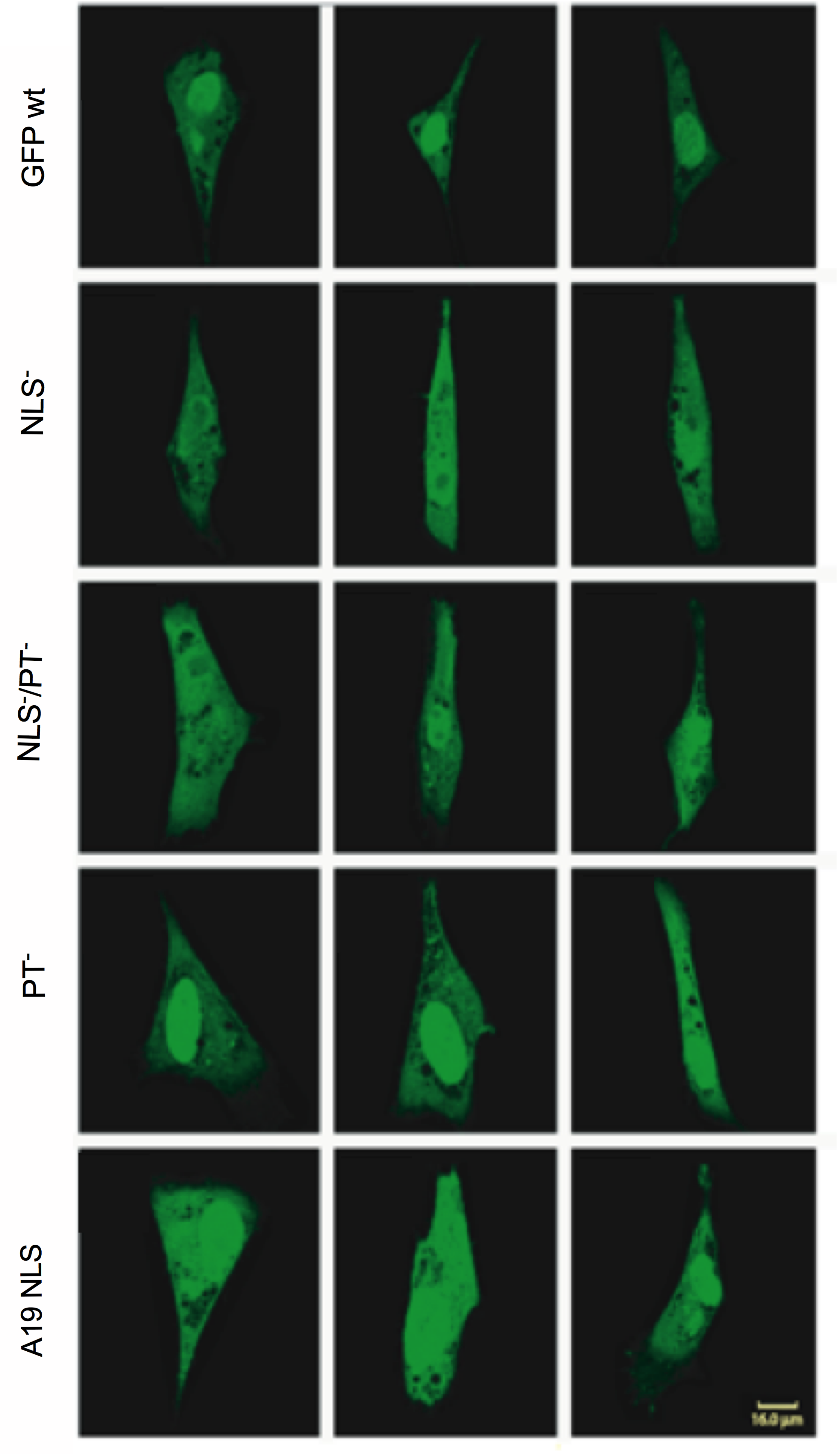

Supplement: Supplementary file 1 [file biomedicines-08-00634-s001.zip › Supplementary_material.pdf]
